# Gonadotropin-Releasing Hormone and Its Role in the Enteric Nervous System

**DOI:** 10.3389/fendo.2017.00110

**Published:** 2017-06-07

**Authors:** Bodil Ohlsson

**Affiliations:** ^1^Lund University, Lund, Sweden; ^2^Division of Internal Medicine, Skåne University Hospital, Lund, Sweden

**Keywords:** enteric nervous system, enteric neurodegeneration, gonadotropin-releasing hormone, gonadotropin-releasing hormone receptor, gonadotropin-releasing hormone antibodies

## Abstract

Gonadotropin-releasing hormone (GnRH), follicle-stimulating hormone, and luteinizing hormone orchestrate the reproduction cycle and regulate the sex steroid secretion from the gonads. In mammals, GnRH1 is secreted as a hormone from the hypothalamus, whereas both GnRH1 and GnRH2 are present as neurotransmitters/peptides in various tissues, where the peptides exert many different effects. mRNA coding for GnRH1 and GnRH2 have been described in the human gastrointestinal tract, and GnRH has been found in both submucosal and myenteric neurons. mRNA coding for GnRH and the fully expressed peptide have been found in rat enteric neurons by some researchers but not by others. mRNA coding for GnRH receptors, but not the fully expressed receptor, has been found in one rat study. GnRH influences gastrointestinal motility and secretion. GnRH analogs are clinically used in the treatment of sex hormone-dependent diseases, i.e., endometriosis and malignancies, and as pretreatment for *in vitro* fertilization. Reduced numbers of enteric neurons and IgM antibodies against GnRH and progonadoliberin-2 (precursor of GnRH2) have been observed after such treatment, with the clinical picture of gastrointestinal dysmotility. Similarly, a rat model of enteric neurodegeneration has been developed after administration of the GnRH analog buserelin. Serum IgM antibodies against GnRH1, progonadoliberin-2, and GnRH receptors have been described in patients with signs and symptoms of gastrointestinal dysmotility and/or autonomic dysfunction, such as irritable bowel syndrome, enteric dysmotility, diabetes mellitus, and primary Sjögren’s syndrome. Thus, apart from regulation of reproduction and sex hormone secretion, GnRH also constitutes a part of enteric nervous system (ENS) and its functions during physiological and pathological conditions. This review aimed to describe the role of GnRH in the ENS.

## Introduction

Gonadotropin-releasing hormone (GnRH) is secreted in a pulsatile fashion from hypothalamic neurons into the portal circulation, where GnRH receptors on the anterior pituitary are activated with subsequent secretion of follicle-stimulating hormone (FSH) and luteinizing hormone (LH) (1, 2). FSH and LH target the gonads and regulate the secretion of steroid hormones (3). Since GnRH is secreted into the portal circulation and has a half-life of a few minutes, the hormone levels cannot be analyzed in peripheral blood (2). Instead, measurements of FSH and LH levels in blood are used to estimate the hypothalamic–pituitary function. In vertebrates, 23 native decapeptides of GnRH exist. Changes of amino acids in molecular positions 5–8 differ the decapeptides from each other (4). In mammals, two types of GnRH have been found: GnRH1 and GnRH2. GnRH1 is secreted from the hypothalamus, whereas both types are present in several organs and tissues of the body, e.g., neural tissue, where they exert neuroendocrine, paracrine, and autocrine functions in the central and peripheral nervous system (4). The GnRH receptor is a G-protein-coupled receptor with seven transmembrane domains (5). Although several different receptors are described, only the GnRH1 receptor is expressed in mammals (3). Both GnRH1 and GnRH2 act through the GnRH1 receptor (4).

## Organization of the Enteric Nervous System (ENS)

The autonomic nervous system is divided into three parts called the sympathetic nervous system, the parasympathetic nervous system, and the ENS (6). The ENS consists of more than 100 million neurons, which are as many neurons as in the spinal cord. The ENS has the ability to control gastrointestinal function independent of brain and spinal cord (7). It is organized in microcircuits, with interneurons and intrinsic afferent neurons, which can initiate reflexes. All kinds of neurotransmitters in the central nervous system (CNS) can be detected in the ENS (7). Nevertheless, 90% of vagal neurons are afferent, suggesting that the brain is mostly a receiver of information (8). The greatest efferent traffic from CNS to the gastrointestinal tract is to the most proximal and most distal parts of the tract, e.g., regulating functions such as mastication and swallowing (7). There is a great evidence that pathophysiological mechanisms in the CNS could also affect the ENS in a similar manner (9).

The ENS consists of two plexus: the myenteric nervous plexus, situated in-between the longitudinal and circular muscle layers, and the submucosal plexus, situated deep in the submucosa. The submucosal plexus mainly regulates the sensory and secretory functions of the gut, whereas the myenteric plexus mainly regulates the motility (7). Both plexus contain excitatory and inhibitory neurotransmitters (7). The submucosal plexus is by unknown reasons less often affected by neurological diseases (9).

## Expression of GnRH and GnRH Receptors in the ENS

Gonadotropin-releasing hormone receptor mRNA was initially described in rat myenteric neurons (10). Later, mRNAs and the fully expressed peptide of GnRH were found in both submucosal and myenteric nerve plexus, whereas GnRH receptors only were found in parasympathetic ganglion cells in rat digestive tract (11). However, neither GnRH nor GnRH receptor could be detected by immunocytochemistry in rat gastrointestinal tract *in vivo* by another research group (12, 13). mRNA for both GnRH1 and GnRH2 could be detected by polymerase chain reaction (PCR) in the human gastrointestinal tract, whereas the GnRH receptor could neither be detected by PCR nor by immunocytochemistry (12) (Table 1). The cellular localization of GnRH has been described in about half of the submucosal and myenteric neurons along the entire human gastrointestinal tract (14, 15).

**Table 1:** **The expression of gonadotropin-releasing hormone (GnRH), GnRH receptor, and luteinizing hormone (LH) receptor in the gastrointestinal tract in rat and humans**.

	GnRH1	GnRH2	GnRH		GnRH receptor	LH receptor
			
	mRNA	mRNA	mRNA	Immunocytochemistry	mRNA	Immunocytochemistry
**Rat**						
Neuron			Huang et al. (11)	Huang et al. (11)	Ho et al. (10)	Sand et al. (12)
						Sand et al. (13)
						Sand et al. (28)
**Human**						
Neuron	Sand et al. (12)	Sand et al. (12)		Ohlsson et al. (14)		Sand et al. (12)
				Hammar et al. (15)		Hammar et al. (47)

*Numbers in brackets are the related references that have described the expression of the mRNA and/or protein*.

## Effects of GnRH or GnRH Analogs on the Function of the ENS

Gonadotropin-releasing hormone and its analog alarelin have been shown to inhibit gastric secretion and gastrin release in rat and dog (Table 2) (16, 17). The mechanisms behind the inhibition seems to be mediated both through direct actions on the parietal cells and by inhibition of the vagus nerve (16, 17). When studying jejunal motility in rats, migrating myoelectric complexes (MMCs) were frequently found during fasted state, albeit more seldom postprandially. After ovariectomy, low-dose treatment of the GnRH agonist leuprolide rendered typical fed-state patterns without MMCs. High-dose treatment of leuprolide inhibited the fed-state pattern and MMCs occurred at a frequency similar to fasted control rats (Table 2). Thus, reproductive hormones have significant effects on gastrointestinal motility (18). However, another GnRH analog could not inhibit the substance P-induced contractions of isolated guinea pig ileum, as it could inhibit substance P-induced elevation of arterial blood pressure (19). Thus, the analog may act as a substance P receptor antagonist in CNS which can inhibit the sympathetic vasomotor outflow, but without effect on peripheral substance P receptors (19).

**Table 2:** **The function of gonadotropin-releasing hormone in the gastrointestinal tract in rat and humans**.

	Rat	Dog	Humans
Gastric secretion	Inhibited (16)	Inhibited (17)	
Gastrin release		Inhibited (17)	
Gastric motility	Modification (18)		Increased (37)
			Reduced (14, 15, 20)
Enteric neuron survival	Reduced *in vivo* and *in vitro* (13, 25–28)		Reduced (14, 15, 20)
Abdominal pain			Increased (21, 22)
			Decreased (29–32)

*Numbers in brackets are the related references*.

## GnRH Analog-Induced Enteric Neurodegeneration in Humans

Pharmacologic treatment with GnRH analogs of endometriosis and pretreatment of *in vitro* fertilization (IVF) has induced severe, gastrointestinal dysmotility in some women (Table 2) (14, 15, 20). Histopathological examination of the patients have revealed a reduced total amount of enteric neurons and a reduced percentage of GnRH-expressing enteric neurons, along with serum IgM antibodies against GnRH1 and/or progonadoliberin-2 (14, 15, 20). Polymorphism in the LH receptor was common in the women who developed severe dysmotility after GnRH treatment (20).

When examining consecutive patients at an infertility clinic, treatment with buserelin led to significantly more symptoms of constipation, nausea and vomiting, impaired psychological well-being, and negative influence of intestinal symptoms on daily life, and a tendency to increased abdominal pain and bloating, compared with prior treatment (21). Five years after the start of the treatment, the patients had increased abdominal pain and better psychological well-being compared with prior IVF treatment. Fifteen percent had developed irritable bowel syndrome (IBS), or had exacerbated symptoms, but none had developed severe dysmotility (21).

In a cohort of women with endometriosis (*n* = 109), patients with a history of GnRH treatment had more severe abdominal pain than patients who had never been treated with GnRH analogs (22). Antibody development seems not to be obligate after GnRH treatment and occurred only in patients developing complications to the treatment (23, 24).

## Buserelin-Induced Enteric Neurodegeneration in Rat

In rat, GnRH-induced enteric neuropathy has been developed after four repeated treatment sessions of buserelin, one session consisting of 5 days of 20 µg daily subcutaneous injections with 3 weeks of recovery (Table 2). This rendered a 50% reduction of both submucosal and myenteric neurons throughout the gastrointestinal tract, although most pronounced in myenteric neurons, and more pronounced distally than proximally in the gastrointestinal tract (13). Signs of ganglionitis were observed (25). Raised serum levels of estradiol, synchronization of the hormonal cycle, and thickened uterine muscle layer point to elevated FSH and LH secretions behind the neurotoxicity (13, 26). Furthermore, a reduced relative number of LH receptor-containing neurons were observed, preceded by increased expression of activated caspase-3 (13). Subclassification of neuron populations in colon showed increased relative numbers of neurons expressing cortiocotropin-releasing factor (CRF) in submucosal neurons and an absolute increased amount of CRF-containing myenteric neurons (27), whereas the relative numbers of neurons expressing calcitonin gene-related peptide, cocaine- and amphetamine-related transcript, galanin, gastrin-releasing peptide, neuropeptide Y, nitric oxide synthase, substance P, vasoactive intestinal peptide, and vesicular acetylcholine transporter were unaffected (26).

An *in vitro* study failed to show any effects on rat enteric neuron survival by the GnRH analog buserelin or by continuous LH stimulation. Instead, intermittent stimulation by a LH analog (lutrotroptin alpha) led to reduced neuronal survival (Table 2) (28).

## Effects of GnRH on Abdominal Symptoms

In a randomized, double-blind, placebo-controlled study of patients with moderate to severe functional bowel disease, continuous treatment with leuprolide during 12 weeks improved symptoms of nausea, vomiting, bloating, abdominal pain, early satiety, and overall gastrointestinal symptoms (Table 2) (29). Continued treatment for 1 year led to even more significant improvements of the symptoms (30). A multicenter study could confirm a significant and persistent improvement in nausea and abdominal pain (31). Leuprolide treatment also improved all gastrointestinal symptoms and quality of life in women with menstrual cycle-related IBS (32).

Two hypotheses to the improved effect by leuprolide on gastrointestinal symptoms in functional bowel disorders have been described (29–32). First, GnRH binds to specific GnRH receptors on the pituitary and controls the secretion of gonadotropins (1). Both LH and ovarian products, such as progesterone and human chorionic gonadotropin (hCG), are neural antagonists of gastrointestinal motility (33, 34). By continuous stimulation of leuprolide, the hypothalamic–pituitary–gonadal axis is downmodulated and the secretion of gonadotropins and gonadal products are inhibited (3, 35). Second, by acting on GnRH receptors on myenteric neurons (10), leuprolide is an effective neural modulator through regulating the voltage-gated calcium channels and the endoplasmic reticulum calcium pump, resulting in the movement and control of intracellular and extracellular calcium (36). However, this assumption is dependent on the presence of fully expressed GnRH receptors in the ENS, which has never been demonstrated at the moment in rat or humans (10–12). Still, peripheral leuprolide restored gastrointestinal motor function both in a transplanted woman who developed chronic intestinal pseudo-obstruction after a virus infection (37) and in female ovariectomized rats (18), whereas administration of the same drug into the intraventricular system of the rat brain had no effect (38).

## Antibody Formation Against GnRH and Gonadotropins

An enzyme-linked immunosorbent assay has been developed to measure GnRH antibodies in serum (14, 21, 39–41). IgM antibodies against GnRH1 have been found in patients with diabetes mellitus, gastrointestinal dysmotility, IBS, posterior laryngitis, and primary Sjögren’s syndrome, independent of treatment with GnRH analogs, in contrast to patients with celiac disease, inflammatory bowel disease, microscopic colitis, and sclerodermia, who express antibodies to the same extent as controls (39–45). IgM antibodies against GnRH receptors have been found in patients with dysmotility, IBS, and primary Sjögren’s syndrome (44, 45), and IgM antibodies against progonadoliberin-2, the precursor of GnRH2 (46), have been found in patients with diabetes mellitus, dysmotility, and IBS (45). Measurements over time showed that the antibody titer in serum was high after each buserelin administration, and the titer was then lowered after some time (14). All patients with reduced number of GnRH-containing enteric neurons displayed IgM antibodies against GnRH1 in serum, independent of GnRH treatment (15).

## Discussion

Gonadotropin-releasing hormone has been found in enteric neurons in both rat and humans by several scientists (11–15). The expression of GnRH receptor is more uncertain, since only one article has described the presence of mRNA for the receptor in rat ENS (10), and no one has demonstrated the fully expressed receptor in submucosal or myenteric plexus of ENS. On the contrary, GnRH receptors have been found in parasympathetic ganglion cells outside the ENS in rat gastrointestinal tract (11), sites not examined in humans. The described effects of GnRH on the ENS are modulation of gastrointestinal motility and secretion (16–19). GnRH treatment has led to enteric neuron death in both rat *in vivo* and *in vitro* trials and in human *in vivo* trials (13–15, 20, 26–28). IgM antibodies against GnRH1, progonadoliberin-2, and GnRH receptors may occur in a subgroup of patients with functional bowel disorders and dysmotility, both in idiopathic forms and when associated with diabetes mellitus, posterior laryngitis, primary Sjögren’s syndrome, or GnRH treatment (39, 40, 42–45).

The GnRH analogs stimulate the anterior pituitary rendering elevated LH secretion with stimulation of the LH receptors and ensuing elevated steroidal sex hormone secretion (1–3, 13, 26). Since the GnRH receptor has not been found in human gastrointestinal tract, the harmful effects evoked on the gastrointestinal tract could be mediated by LH receptors, which are found in the gastrointestinal tract in humans and rat (12, 13, 28, 47), and are downregulated after GnRH stimulation (13). Both LH and hCG exert their effects through LH receptors. As well, LH, hCG, and progesterone are known to reduce gastrointestinal motility (33, 34), which could explain the reduced symptom burden after continuous GnRH stimulation due to downregulated secretion of gonadotropins and sex steroids (1, 2, 29–32). The effects evoked by LH receptor stimulation seem to be mediated through cAMP/protein kinase A. Furthermore, LH stimulation leads to a change in gene transcripts coding for steroidogenic enzymes, cytoskeletal proteins, in addition to signaling molecules coding for pro- and antiapoptotic processes (48). A downregulation of LH receptors is therefore accompanied by decreased apoptosis (49), which was reflected by the increased relative number of activated caspase-3 immunoreactive enteric neurons prior to the neuronal loss in the GnRH-induced rat model of neuropathy (13). *In vitro* trials on enteric rat neurons confirmed this theory, with a reduced neuron survival only after intermittent stimulation with the LH analog lutrotropin alpha, and not with GnRH analogs (28).

*In vitro* fertilization treatments leads to repeated unphysiological LH stimulation, which may be the cause of severe dysmotility observed in some women with a polymorphism in the LH receptor (20). The LH receptor is present in both genital organs and the gastrointestinal tract (12, 13, 28, 47, 50) and could be a plausible explanation to the observed association in women between dysfunction of the digestive tract and diseases of genital organs (18, 51, 52). As much as 50% loss of enteric neurons were accompanied with mostly normal gastrointestinal function (26, 27), suggesting a huge reserve capacity of the ENS. Thus, full-thickness biopsies are mandatory to examine the effects of GnRH treatment on the ENS (13–15, 26, 27) and to differ functional bowel symptoms from enteric dysmotility.

The fact that some research groups have been able to demonstrate GnRH and its receptor mRNA in the rat ENS (10, 11), while not found by others (12, 13), may have several reasons. The native GnRH receptor could not be found in adult rat neurons from the superior cervical ganglion (53). However, after microinjection of cRNA coding for the human GnRH receptor, the expression of the protein could be demonstrated (53). Thus, the expression of GnRH receptors in the neural tissue may vary. Although the GnRH receptor has not been able to demonstrate, it can still be present in the ENS. In addition to a central stimulation by GnRH administration, GnRH may thus also exert peripheral effects, direct on the ENS. In rat hypothalamus, a cross talk between *N*-methyl-d-aspartate and adrenergic neurotransmission has been demonstrated in the regulation of the hypothalamic GnRH gene expression (54). Due to release of nitric oxide from endothelial cells, the vascular endothelium is involved in the release of neurohormones from the median eminence (55). Theoretically, similar cross talks and mediators from the epithelial cells may be present in the ENS, which has not been examined at all regarding the release and regulation of GnRH and LH and their receptors. Thus, we are in the beginning of this research field, and experimental *in vivo* and *in vitro* trials are necessary to further determine the expression and function of these peptides in the ENS and digestive tract. If GnRH receptors are present in the ENS, the same mechanisms may be involved in the GnRH-induced response in ENS as in the gonadotrophs, e.g., activation of cAMP, cGMP, phospholipases, and calcium channels (3). It has been found that subjects with autonomic dysfunction had an abnormal hypothalamic gonadotrophin secretion (56). The mechanisms involved in the regulation of GnRH, LH, and their receptors in subjects with dysfunction of the ENS have not been determined to date.

Although chronic GnRH treatment may improve abdominal symptoms, these analogs are not used in the clinical setting, due to the risk of menopausal symptoms and development of osteoporosis in long-term treatment (57). During the last years, stimulation with GnRH agonists has been replaced by administration of GnRH antagonists in the IVF setting, to prevent the LH surges and thereby to reduce the side effects (2). The observation of more abdominal pain in endometriosis patients with GnRH treatment may reflect that GnRH analogs in this treatment group induce enteric neuropathy with ensuing abdominal pain, apart from endometriosis pain (22). Even if the GnRH analog-treated endometriosis patients are the patients with most severe disease and pain, and the elevated pain in this group could reflect more severe disease, GnRH treatment seems not to be efficient to reduce pain.

Antibodies against neuronal tissue have previously been described secondary to gut dysmotility (58). GnRH antibodies may represent neuronal damage in a subgroup of patients and may not be causal, since GnRH analogs *per se* did not induce serum antibody expression in humans or rat (13, 21, 26), and antibodies were present also in patients without previous GnRH treatment (14, 15, 20, 39, 40, 42–45). The absence of GnRH antibodies in rats may depend on lack of GnRH expression in rat enteric neurons (13, 26), or very small amounts of the peptide (10, 11). GnRH1 and GnRH2 are present in both the central and peripheral nervous system (3, 4, 12, 46). Autonomic neuropathy and gastrointestinal complaints are common in patients with diabetes mellitus and primary Sjögren’s syndrome (42, 44) and autonomic neuropathy, depression, and affective disorders are common in patients with functional bowel diseases and gastrointestinal dysmotility (58–61). Theoretically, antibodies against GnRH may be secondary to either a central neuronal damage or a peripheral neuronal damage (39, 40, 42, 44, 45).

## Conclusion

Gonadotropin-releasing hormone has been found in the human ENS in repeated examinations. GnRH has been found in rat ENS in some studies, although not reproducible by others. Fully expressed GnRH receptors have never been found in rat or human ENS. GnRH modulates gastrointestinal motility and secretion. Treatment with GnRH analogs may induce enteric neurodegeneration in both rat and human ENS. LH receptor activation is the postulated target of the effects observed, since LH receptors are described on enteric neurons and enteric rat neuronal survival was decreased after intermittent *in vitro* stimulation of the LH receptor. Autoantibodies against GnRH and its receptor are found in a subgroup of patients with disturbances from the gastrointestinal tract and/or autonomic nervous system, independent of treatment with GnRH analogs.

Altogether, the knowledge about GnRH expression and function in the gastrointestinal tract suggests a role for GnRH on the ENS, but the field is rudimentary studied. The key point in this research field is to study the local effect of GnRH in the gastrointestinal tract and the communication between GnRH and LH receptors. Future research should include a search for fully expressed GnRH receptors in rat and human ENS. Further, a controlled study comparing the effect of GnRH analogs compared with GnRH antagonists on neuron survival should be performed *in vitro* and *in vivo*. Another alternative is to compare the effects on the ENS by sole GnRH analogs and GnRH analogs in combination with LH receptor antagonists. Both cell culture trials with enteric neurons and organ bath experiments are needed to characterize the route of effects by GnRH on the ENS, and the effects evoked.

## Author Contributions

The author confirms being the sole contributor of this work and approved it for publication.

## Conflict of Interest Statement

The author declares that the research was conducted in the absence of any commercial or financial relationships that could be construed as a potential conflict of interest.

## Abbreviations

ELISA, enzyme-linked immunosorbent assay; FSH, follicle-stimulating hormone; GnRH, gonadotropin-releasing hormone; hCG, human chorionic gonadotropin; IBS, irritable bowel syndrome; IVF, *in vitro* fertilization; LH, luteinizing hormone; MMC, migrating myoelectric complex; PCR, polymerase chain reaction.
